# Changes in Sleep Patterns and Disorders in Children and Adolescents with Attention Deficit Hyperactivity Disorders and Autism Spectrum Disorders during the COVID-19 Lockdown

**DOI:** 10.3390/brainsci11091139

**Published:** 2021-08-27

**Authors:** Oliviero Bruni, Maria Breda, Raffaele Ferri, Maria Grazia Melegari

**Affiliations:** 1 Department of Developmental and Social Psychology, Sapienza University, 00185 Rome, Italy; mgraziamelegari@gmail.com; 2 Child Neurology and Psychiatry Unit, Department of Human Neurosciences, Sapienza University, 00185 Rome, Italy; maria.breda@uniroma1.it; 3 Sleep Research Centre, Department of Neurology IC, Oasi Research Institute—IRCCS, 94018 Troina, Italy; rferri@oasi.en.it; 4 Consortium “Humanitas” LUMSA University, 00193 Rome, Italy

**Keywords:** sleep, ADHD, ASD, COVID-19

## Abstract

Background. The COVID-19 lockdown determined important changes in the sleep of a large percentage of the world population. We assessed the modifications of reported sleep patterns and disturbances in Italian children and adolescents with autism spectrum disorders (ASD) or attention deficit hyperactivity disorders (ADHD), compared to control children, before and during the COVID-19 lockdown in Italy. Methods. Parents of 100 ASD, 236 ADHD patients, and 340 healthy children filled out an anonymous online survey and a modified version of the Sleep Disturbance Scale for Children (SDSC), advertised via social media, to evaluate sleep patterns and disturbances of their children before and during the lockdown. Results. Before the lockdown, bedtime and risetime were not different between the three groups. During the lockdown, ADHD children tended to have a later bedtime and risetime than ASD and controls, while ASD children tended to maintain similar bedtime and risetime. Overall, during the lockdown, a reduced sleep duration significantly differentiated clinical groups from controls. Anxiety at bedtime, difficulties in falling asleep, and daytime sleepiness increased in all groups during the lockdown. Hypnic jerks, rhythmic movement disorders, night awakenings, restless sleep, sleepwalking, and daytime sleepiness increased in ASD and ADHD patients, in particular. Conclusions. This is the first study comparing sleep habits and disorders in ASD and ADHD during the lockdown showing specific differences consistent with the core characteristics of two neurodevelopmental disorders.

## 1. Introduction

The general confinement from the COVID-19 pandemic and the consequent school closure, interruption of contacts with family members and friends, as well as reduced social and leisure activities, has brought unparalleled modifications to the lifestyle of children and adolescents, with important repercussions on their mental state and behaviors [1].

In particular, COVID-19 lockdown resulted in important changes in sleep habits and sleep disorders in a large portion of the world’s population of all ages [2,3,4,5,6]. The increase in sleep difficulties was often associated with higher levels of psychopathological symptoms or decreased quality of life [3,7,8].

In face of a great amount of literature on the young and adult general population, only a few studies were conducted on sleep patterns or disturbances of the clinical population, especially children and adolescents with autism spectrum disorder (ASD) [9] and attention-deficit/hyperactivity and impulsive disorders (ADHD) [10], despite their frequent sleep disturbances and altered sleep patterns, independently by COVID-19 [11]. Since sleep patterns significantly changed during the lockdown in typically developing children and adolescents [2,5,6,12], similar or even worse changes in subjects with ADHD and ASD could be expected. Several studies [9,10,13,14,15,16,17] reported a high percentage of patients with ASD or ADHD that changed their sleep with a significant worsening of sleep quality and disturbances during the lockdown. Specifically, ASD children showed significant worsening of sleep disturbances, sleep duration, and sleep quality [14,15] and an increase in bedtime resistance, delay in falling asleep, and night awakenings [17]. Children with ADHD showed an alteration of sleep patterns in 85% of cases [16] and, unlike their healthy peers in the control group that experienced an increase in school night sleep duration, they did not benefit from the COVID-19 lockdown [13].

Significant changes in sleep habits and disturbances in children with ADHD or ASD and adolescents during the lockdown have been reported by other studies. Since ADHD and ASD are two neurodevelopmental disorders with different symptoms, we could have expected that sleep changes would reflect specific clinical traits characterizing these two populations. To the best of our knowledge, no study has compared these two disorders for their sleep patterns and disturbances during the COVID-19 lockdown, thus, the aim of this study was to evaluate the different responses of these two clinical conditions in terms of sleep patterns and sleep disturbances. 

## 2. Materials and Methods 

### 2.1. Participants

Parents of Italian children and adolescents completed an online survey, advertised via social media, for a limited time window (from 7th May to 15th June 2020), targeting children aged 4 to 18 years. ASD and ADHD children and adolescents had been diagnosed by a child and adolescent psychiatrist of the Child and Adolescent Mental Health Services, before the survey, and were being followed at the same center. The survey was developed and conducted following the guidelines set by the Checklist for Reporting Results of Internet E-Surveys (CHERRIES) [18]. From a total of 5825 respondents, we identified ADHD and ASD patients and selected randomly a sample of typically developing subjects matched for age and sex with the clinical groups.

In total, 100 ASD (16 females, 16% and 84 males, 84%), 236 ADHD patients (44 females, 18.6% and 192 males, 81.4%), and 340 controls (58 female, 17.1% and 282 males, 82.9%) were enrolled in the analysis. 

In Italy, the COVID-19 lockdown started on March 2020 with important restrictions that included school closure, limited activities for businesses and factories, and movement restrictions. The strict lockdown in Italy lasted for almost three months, with the progressive reopening of several activities in mid-June 2020. Italian schools reopened in September 2020, with some limitations.

There was no monetary or credit compensation for participating in the study. The study protocol was approved by the Ethics Committee of the Department of Developmental and Social Psychology of the Sapienza University of Rome and was conducted in accordance with the Declaration of Helsinki. 

### 2.2. Measures

A specific questionnaire was arranged for the survey. The first section was devoted to the collection of demographic data (age, gender, caregiver education, region of Italy). A second section was organized to gather information on sleep arrangement and schedule during weekdays and during weekends (bedtime, risetime, sleep latency, sleep duration, co-sleeping). All these questions were asked in order to evaluate differences between before and during the lockdown period. A third section of the survey was related to family composition, work of parents during the lockdown, online lessons for children and adolescents, screen exposure time (excluding the hours for lessons), use of over-the-counter or prescription drugs for sleep. Caregivers completed a modified version of the Sleep Disturbance Scale for Children (SDSC) [19]. 

Retrospective questions were used to estimate perceived changes across two time periods: from “before the lockdown” (i.e., in the last month before the outbreak) to “during the lockdown” (i.e., in the seven days prior to filling out the survey). 

The SDSC was originally validated on a sample of 6- to 16-year-old healthy children from the general population [19] but was also used for younger children [20,21]. We grouped questions related to sleep-disordered breathing into one question and selected, in total, 13 items in order to facilitate the compilation by parents.

### 2.3. Data Analysis

Descriptive statistics were applied to characterize sociodemographic variables, sleep patterns, and sleep disturbances. Data were reported as frequencies and percentages for comparisons between the groups. The McNemar’s test was performed to compare sleep patterns and sleep disturbances before and during the lockdown. “Before-during” bedtime and risetime were recorded within three categories based if maintained, delayed, or anticipated and sleep duration if maintained, increased, or reduced. Chi-square tests were conducted to compare changes in sleep patterns, sleep schedule, and sleep disturbances before and during lockdown, within and between the groups. Fisher’s exact test was applied when appropriate. 

For all comparisons, *p*-values less than 0.05 were considered to be statistically significant. Statistical analyses were performed using the SPSS software release 17.0 (SPSS INC, Chicago, IL, USA).

## 3. Results

Demographics of the sample are reported in Table 1.

In all three groups, there was a prevalence of males. Most of the parents providing data on their child’s sleep habits were mothers and most families had a middle income.

### 3.1. Comparison of Sleep Patterns in the Three Groups before and during Lockdown

#### 3.1.1. Bedtime

**Figure 1:**
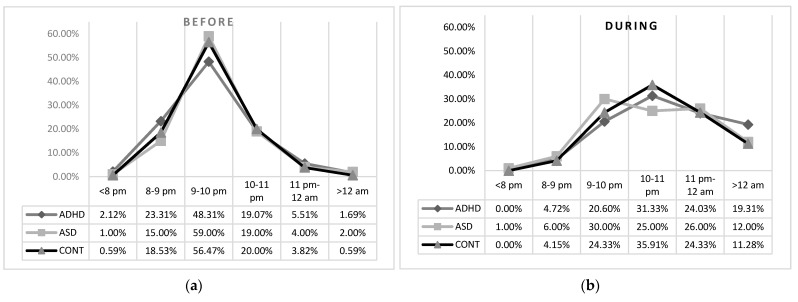
Bedtime before (**a**) and during (**b**) lockdown in the tree groups.

Before lockdown, no differences were found for bedtime during weekdays in the three groups. During lockdown, no differences were found between the two clinical groups, but the ADHD group that had a higher percentage of children going to bed after 12 a.m. than controls and the ASD group had a lower percentage of children going to bed between 10 to 11 p.m. than controls (Table 2; Figure 1). 

#### 3.1.2. Risetime

**Figure 2:**
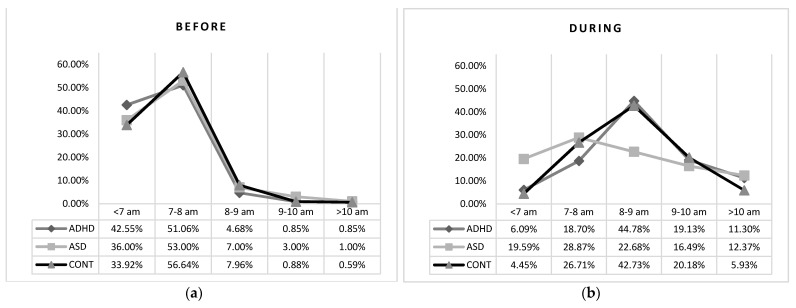
Risetime before (**a**) and during (**b**) lockdown in the tree groups.

Similarly, before lockdown, no differences were found for risetime during weekdays, probably due to common school schedules (Table 3; Figure 2).

During lockdown, the lack of obliged risetime led to the appearance of important differences: 19.6% of ASD children continued to wake up before 7 a.m., whereas only 6.1% of ADHD and 4.45% of the control group stayed in this time slot. Most ADHD children and controls (44.8% and 42.8%, respectively) woke up between 8 and 9 a.m., whereas ASD children’s risetime during lookdown was more uniformly distributed in the early morning hours (Table 3; Figure 2). Both clinical groups had a significantly higher percentage of children with risetime after 10 a.m. than controls.

#### 3.1.3. Sleep Duration

**Figure 3:**
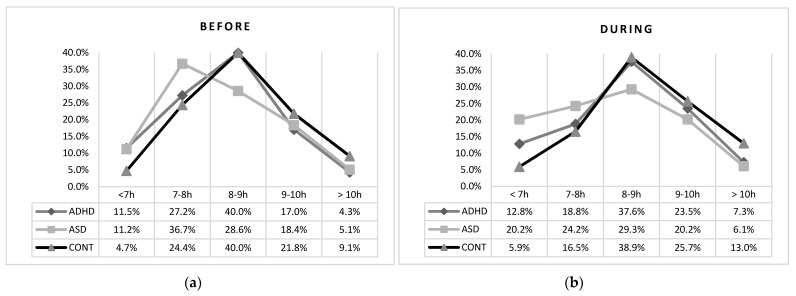
Sleep duration before (**a**) and during (**b**) lockdown in the tree groups.

Before lockdown, both clinical groups, when compared to controls, had a significantly higher percentage of children sleeping less than 7 h (Table 4, Figure 3). Moreover, the ASD group showed a lower percentage of children sleeping between 8 to 9 h/night than the ADHD and control groups. 

During lockdown, no differences in sleep duration were found between the two clinical groups while both clinical groups had a significantly higher percentage of children sleeping less than 7 h than controls (Table 4; Figure 3).

### 3.2. Comparison of Sleep Patterns during Lockdown

ADHD children showed a higher percentage of delaying and a lower percentage of maintaining weekday bedtime than ASD and controls and tended to delay risetime during weekday and weekend more than ASD but not than controls (Table 5). Sleep duration changes were not different between the ADHD and ASD groups, while controls showed a higher percentage of children who maintained the same sleep duration. Compared to controls, both clinical groups increased the weekday and weekend sleep latency during the lockdown (Table 6).

### 3.3. Comparison of the Prevalence of Sleep Disorders in the Three Groups before and during Lockdown

#### 3.3.1. Intragroup Comparison

During lockdown, we observed an increased prevalence of several sleep disorders, especially in the two clinical groups (Supplementary Table S1). 

A significant increase in anxiety at bedtime and daytime sleepiness was found in all the three groups: anxiety at bedtime increased from 15.3% to 23.7% in ADHD children (*p* = 0.007), from 12.0% to 22.0% in the ASD group (*p* = 0.006), and from 5.6 to 11.2% in controls (*p* = 0.003).

Difficulty in falling asleep increased in the two clinical groups: from 27.1% to 39.0% in the ADHD group (*p* = 0.001), and from 23.0% to 35.0% in the ASD group (*p* = 0.029). 

Prevalence of sleepwalking increased significantly only in the ADHD and control groups, from 0.4% to 5.9% (*p* = 0.000) for ADHD and from 0.0% to 2.4% (*p* = 0.000) for controls. 

Nightmares increased in all the three groups but significantly only in controls, from 4.7% to 10.3% (*p* = 0.003). Daytime sleepiness increased from 12.3% to 19.9% in the ADHD group (*p* = 0.005), from 4.0% to 15.0% in the ASD group (*p* = 0.003) and from 4.4% to 7.9% in the control group (*p* = 0.029). 

The only significant decrease in sleep disorders was found for bruxism in ADHD children that diminished from 16.5% to 11.0% (*p* = 0.015).

#### 3.3.2. Intergroup Comparison

While before lockdown there was no difference between the clinical groups and controls, during lockdown difficulties in falling asleep significantly increased in the two clinical groups resulting in a significant difference with the control group. 

Anxiety at bedtime, hypnic jerks, rhythmic movement disorder, and night awakenings were found to be significantly higher in the clinical groups than in controls, both before and during lockdown. 

Restless sleep and snoring/apnea were significantly higher in both clinical groups than in controls (in ASD, only during lockdown) (Table 7).

Compared to controls, sleepwalking was more prevalent in the ASD group, both before and during lockdown, while sleep terrors were more prevalent only during lockdown. 

The prevalence of bruxism and nightmares was significantly increased in children with ADHD compared to ASD and controls, both before and during lockdown. 

On the other hand, no differences were found between ASD and controls in the prevalence of nightmares and bruxism (Table 7).

Daytime sleepiness was higher in ADHD than in control children, both before and during lockdown, and slightly more prevalent in ASD than in controls during lockdown (Table 7; Figures S1 and S2).

## 4. Discussion

The lockdown experience significantly impacted sleep patterns and disturbances of children and adolescents with ADHD and ASD, as well as of controls. As also indicated by other studies [5,6,12], sleep is one of the more impaired domains during the COVID-19 lockdown, independently by mental health condition and age and it is known that sleep problems in ASD and ADHD patients may worsen daytime behavior and functioning, as well as increase parental distress [11]. We found, in both clinical groups, a higher percentage of subjects that reported a reduced sleep duration and an increased sleep latency than controls. ASD patients showed less changes in weekday-weekend risetime than both ADHD and controls, while children with ADHD reported higher delays in bedtime than the other groups. Our findings are supported by specific studies on these two clinical groups and controls [2,16], reporting delayed bedtime in children with ADHD [10,17] and a reduced sleep duration in both ADHD and ASD [9,10,13,15,22].

Although changes of lifestyle caused by the lockdown affected both clinical groups and controls, generally children and adolescents with ADHD showed greater instability of their sleep schedule and increased delay in weekday sleep schedule, when compared to ASD and controls, during lockdown. This finding, according to the night-to-night variability in the sleep–wake patterns reported by several comparative studies in children with ADHD compared to typically developing children [23,24,25], supports the consideration that the “variability” of sleep patterns, represents a distinctive marker of the ADHD condition.

Since ADHD patients’ functioning is strongly dependent on environmental changes, in agreement with other studies [10,26], our findings confirm that sudden lifestyle changes caused by the pandemic lockdown impacted the sleep and behavior of ADHD patients more than on those of ASD patients or controls. Conversely, children and adolescents with ASD seemed to be less vulnerable to the effects of prolonged isolation, showing a higher stability in bedtime and risetime than both ADHD and controls, probably linked to the stereotyped and fixed behavior of these children, since inflexibility and insistence on sameness are hallmark characteristics of ASD [15].

The significantly decreased sleep duration in the ADHD group, compared to controls, is supported by the study by Becker et al. [13], suggesting that ADHD patients did not benefit from the COVID-19 lockdown, unlike their healthy peers of the control group that experienced an increase in school days night sleep duration and were more likely to obtain recommended sleep duration during COVID-19. Similarly, Mutluer et al. [15] reported a decrease in the number of hours ASD children slept from before to during COVID-19. Finally, our findings agree with those by Bruni et al. [2] who reported a general stable sleep duration among healthy children, with only a small but significant increase during lockdown.

We found an increase in several sleep disorders during the lockdown: some disorders increased in all the three groups, such as anxiety at bedtime and daytime sleepiness while difficulties in falling asleep hypnic jerks, rhythmic movement disorder, night awakenings, restless sleep, sleepwalking, and daytime sleepiness increased especially in patients with ASD and ADHD. Interestingly, at baseline, ADHD children resulted to have increased bruxism, nightmares, and daytime sleepiness than their ASD peers, while during the lockdown ASD and ADHD children and adolescents had a similar prevalence of all sleep disturbances, with the exception of nightmares that were more frequent in ADHD children. 

Very few studies have been published on the impact of the COVID-19 lockdown on sleep disorders of patients with ASD and ADHD. Regarding the sleep disorders of ASD patients during the pandemic, our results are in agreement with those by Türkoğlu et al. [17] that reported an increase in difficulties (and delay) in falling asleep, and anxiety at bedtime; in contrast, we did not find an increase in night awakenings, but we observed an increase in daytime sleepiness. Similarly, Lugo-Marin et al. [14] reported a deterioration in sleep quality in 56% of 100 ASD patients (children and adults). Very few studies have examined sleep disorders of ADHD youths during COVID-19. In a sample of 241 youth (aged 6–15 years) with ADHD in China, 48% of parents indicated that COVID-19 had not changed their child’s sleep, with 20% indicating that sleep had worsened, and 32% indicating that sleep had improved. Becker et al. [13] reported less improvement of daytime sleepiness in ADHD adolescents compared to their healthy peers of the control group. 

Several sleep symptoms that we found increased in our ASD and ADHD children have been recognized to be an expression of a psychological distress condition [27,28] strictly related to the pandemic.

Altogether, our findings confirm a great distress susceptibility in both clinical children due to the sudden changes of lifestyle imposed by the lockdown experience that determined a vulnerability of their sleep–wake patterns. The different sleep pattern alterations in the two clinical groups might be linked to their core traits defining two distinct disorders: children with ASD, that tended to maintain their sleep–wake schedule, were less vulnerable due to the stereotyped behavior and insistence on sameness while children with ADHD, that showed greater changes in sleep–wake patterns, were more vulnerable, due to their innate behavior instability, swift changes in mood, and low frustration tolerance. 

We have to consider that the interruption of social relationships, the reduction in physical activities as well as the academic and normal working activities, had an impact on sleep. In parallel, other studies showed a strong impact of lockdown on affective domains, highlighting mainly an increase in depression symptoms (for ADHD see Melegari et al., 2021) [26]. Further studies focused on the lockdown impact on both sleep patterns and affective states in these clinical populations should contribute to explain their reciprocal interaction. Some limitations of this study need to be considered, such as the sample size (although relatively large for this condition) and sampling only respondents from a single country; thus, results may not be fully generalizable to other countries. In addition, the predetermined sample size to adequately reduce the risk of type II error was not achieved. Furthermore, most subjectively reported elements of the SDSC should be interpreted with caution due to potential parental misinterpretation in ASD subjects that are often non-verbal. Although the survey was conducted after only a few days from the end of the strict lockdown and in the presence of lighter restrictions, we cannot exclude a memory bias of the parents. Finally, we should consider that self-selection bias is inherent with the online survey methodology employing nonprobability sampling. On the other hand, a strength of the study is that examined similarity or differences in sleep pattern changes using the same instruments and the same temporal window of investigation.

## 5. Conclusions

To our knowledge, this is the first study comparing sleep habits and disorders in two neurodevelopmental disorders, ASD and ADHD, highlighting the differences in sleep patterns and disorders between these two different clinical populations, both at baseline and during the COVID-19 lockdown. It will be important to examine whether changes in sleep persist over time, continue to change, or return to the pre-COVID-19 levels as the pandemic continues.

## Figures and Tables

**Table 1:** Demographics of the three groups of children enrolled in this study.

Gender	ADHD	ASD	Controls
**Total**	236	100	340
**Gender**			
F	44 (18.6%)	16 (16.0%)	58 (17.1%)
M	192 (81.4%)	83 (83.0%)	282 (82.9%)
**Age**			
4–5 years	23 (9.7%)	24 (24.0%)	57 (16.8%)
6–12 years	155 (65.7%)	47 (47.0%)	192 (56.5%)
13–18 years	58 (24.6%)	29 (29.0%)	91 (26.8%)
**Respondent**			
Mother	221 (93.6%)	91 (91.0%)	315 (92.6%)
Father	14 (5.9%)	9 (9.0%)	3 (0.9%)
Grandparent	1 (0.4%)	0 (0.0%)	22 (6.5%)
**Education level of respondent**			
Graduation	79 (33.6%)	41 (41.0%)	169 (49.9%)
High schools	123 (52.3%)	49 (49.0%)	142 (41.9%)
Middle schools	27 (11.5%)	9 (9.0%)	26 (7.7%)
Elementary schools	6 (2.6%)	1 (1.0%)	2 (0.6%)
**Family income**			
Low	45 (19.2%)	17 (17.0%)	45 (13.5%)
Middle	183 (78.2%)	79 (79.0%)	258 (77.2%)
High	6 (2.6%)	4 (4.0%)	31 (9.3%)
**Siblings**			
Only child	80 (34.0%)	38 (38.4%)	86 (25.3%)
2 children	128 (54.5%)	45 (45.5%)	191 (56.2%)
3 children	24 (10.2%)	14 (14.1%)	51 (15.0%)
≥4 children	3 (1.3%)	2 (2.0%)	12 (3.5%)

**Table 2:** Comparison between bedtime in weekdays before and during lockdown in the three groups.

				ADHD vs. ASD		ADHD vs. Controls		ASD vs. Controls	
	ADHD	ASD	Controls	*χ^2^*	*p*	*χ^2^*	*p*	*χ^2^*	*p*
**Bedtime WD before**									
<8 p.m.	5 (2.1%)	1 (1.0%)	2 (0.6%)	-	NS *	-	NS *	-	NS *
8–9 p.m.	55 (23.3%)	15 (15.0%)	63 (18.5%)	2.937	NS	1.950	NS	0.660	NS
9–10 p.m.	114 (48.3%)	59 (59.0%)	192 (56.5%)	3.216	NS	3.730	NS	0.202	NS
10–11 p.m.	45 (19.1%)	19 (19.0%)	68 (20.0%)	0.000	NS	0.077	NS	0.049	NS
11 p.m.–12 a.m.	13 (5.5%)	4 (4.0%)	13 (3.8%)	-	NS *	0.918	NS	-	NS *
>12 a.m.	4 (1.7%)	2 (2.0%)	2 (0.6%)	-	NS *	-	NS *	-	NS *
**Bedtime WD during**									
<8 p.m.	0 (0.0%)	1 (1.0%)	0 (0.0%)	-	NS *	-	NS	-	NS *
8–9 p.m.	11 (4.7%)	6 (6.0%)	14 (4.2%)	0.236	NS	0.106	NS	0.602	NS
9–10 p.m.	48 (20.6%)	30 (30.0%)	82 (24.3%)	3.446	NS	1.090	NS	1.330	NS
10–11 p.m.	73 (31.3%)	25 (25.0%)	121 (35.9%)	1.350	NS	1.284	NS	4.122	**0.042**
11 p.m.–12 a.m.	56 (24.0%)	26 (26.0%)	82 (24.3%)	0.146	NS	0.007	NS	0.115	NS
>12 a.m.	45 (19.3%)	12 (12.0%)	38 (11.3%)	2.638	NS	7.153	**0.007**	0.040	NS

WD = weekday. Significant differences at *p* < 0.05 are in bold. * Fisher’s exact test was applied.

**Table 3:** Comparison between risetime in weekdays before and during lockdown in the three groups.

				ADHD vs. ASD		ADHD vs. Controls		ASD vs. Controls	
	ADHD	ASD	Controls	*χ^2^*	*p*	*χ^2^*	*p*	*χ^2^*	*p*
**Risetime WD before**									
<7 a.m.	100 (42.6%)	36 (36.0%)	115 (33.9%)	1.249	NS	4.412	**0.036**	0.148	NS
7–8 a.m.	120 (51.1%)	53 (53.0%)	192 (56.6%)	0.105	NS	1.738	NS	0.414	NS
8–9 a.m.	11 (4.7%)	7 (7.0%)	27 (8.0%)	0.742	NS	2.421	NS	0.101	NS
9–10 a.m.	2 (0.9%)	3 (3.0%)	3 (0.9%)	-	NS *	-	NS *	-	NS *
> 10 a.m.	2 (0.9%)	1 (1.0%)	2 (0.6%)	-	NS *	-	NS *	-	NS *
**Risetime WD during**									
<7 a.m.	14 (6.1%)	19 (19.6%)	15 (4.5%)	13.70	**0.000**	0.754	NS	23.901	**<0.001**
7–8 a.m.	43 (18.7%)	28 (28.9%)	90 (26.7%)	4.152	**0.042**	4.886	**0.027**	0.177	NS
8–9 a.m.	103 (44.8%)	22 (22.7%)	144 (42.7%)	14.11	**<0.001**	0.234	NS	12.891	**<0.001**
9–10 a.m.	44 (19.1%)	16 (16.5%)	68 (20.2%)	0.316	NS	0.095	NS	0.655	NS
>10 a.m.	26 (11.3%)	12 (12.4%)	20 (5.9%)	0.076	NS	5.287	**0.021**	4.569	**0.033**

WD = weekday. Significant differences at *p* < 0.05 are in bold. * Fisher’s exact test was applied.

**Table 4:** Comparison between sleep duration in weekdays before and during lockdown in the three groups.

				ADHD vs. ASD		ADHD vs. Controls		ASD vs. Controls	
	ADHD	ASD	Controls	*χ^2^*	*p*	*χ^2^*	*p*	*χ^2^*	*p*
**Sleep duration WD before**									
<7 h	27 (11.5%)	11 (11.2%)	16 (4.7%)	0.005	NS	9.241	**0.002**	5.588	**0.018**
7–8 h	64 (27.2%)	36 (36.7%)	83 (24.4%)	2.971	NS	0.582	NS	5.838	**0.016**
8–9 h	94 (40.0%)	28 (28.6%)	136 (40.0%)	3.891	**0.049**	0.000	NS	4.242	**0.039**
9–10 h	40 (17.0%)	18 (18.4%)	74 (21.8%)	0.087	NS	1.967	NS	0.529	NS
> 10 h	10 (4.3%)	5 (5.1%)	31 (9.1%)	0.115	NS	4.961	0.026	1.626	NS
**Sleep duration WD during**									
<7 h	30 (12.8%)	20 (20.2%)	20 (5.9%)	2.971	NS	8.326	**0.002**	18.888	**<0.001**
7–8 h	44 (18.8%)	24 (24.2%)	56 (16.5%)	1.266	NS	0.501	NS	3.062	NS
8–9 h	88 (37.6%)	29 (29.3%)	132 (38.9%)	2.110	NS	0.104	NS	3.066	NS
9–10 h	55 (23.5%)	20 (20.2%)	87 (25.7%)	0.435	NS	0.347	NS	1.238	NS
>10 h	17 (7.3%)	6 (6.1%)	44 (13.0%)	0.157	NS	4.752	**0.029**	3.627	NS

WD = weekday. Significant differences at *p* < 0.05 are in bold.

**Table 5:** Comparison of weekday–weekend bedtime and risetime in the three groups.

	Delayed	Advanced	Maintained	*χ^2^*	*p*	*χ^2^*	*p*	*χ^2^*	*p*
**Bedtime WD**									
ADHD	188 (80.7%)	0	45 (19.3%)	19.947	**<0.001**	9.824	**0.007**	4.845	NS
ASD	58 (58%)	1 (1%)	41 (41%)						
Controls	235 (69.7%)	3 (0.9%)	99 (29.4%)						
**Bedtime WE**									
ADHD	98 (42.4%)	5 (2.2%)	128 (55.4%)	2.280	NS	5.621	NS	1.875	NS
ASD	44 (44.4%)	5 (5.1%)	50 (50.5)						
Controls	124 (36.8%)	20 (5.9%)	193 (57.3%)						
**Risetime WD**									
ADHD	192 (81.4%)	3 (1.3%)	34 (14.8%)	22.444	**<0.001**	6.602	NS	20.606	**<0.001**
ASD	59 (60.8%)	7 (7.2%)	31 (32%)						
Controls	256 (76.2%)	2 (0.6%)	78 (23.2%)						
**Risetime WE**									
ADHD	136 (58.6%)	41 (17.7%)	55 (23.7%)	33.333	**<0.001**	2.199	NS	43.600	**<0.001**
ASD	31 (31%)	13 (13%)	56 (56%)						
Controls	216 (63.9%)	46 (13.6%)	76 (22.5%)						

WD = weekday; WE = weekend. Significant differences at *p* < 0.05 are in bold.

**Table 6:** Comparison of weekday–weekend sleep duration and sleep latency in the three groups.

				ADHD vs. ASD		ADHD vs. Controls		ASD vs. Controls	
	Increased	Decreased	Maintained	*χ^2^*	*p*	*χ^2^*	*p*	*χ^2^*	*p*
**Sleep duration WD**									
ADHD	80 (34.3%)	59 (25.3%)	94 (40.3%)	5.318	NS	16.165	**<0.001**	7.214	**0.027**
ASD	22 (22.4%)	25 (25.5%)	51 (52%)						
Controls	101 (29.8%)	49 (14.4%)	189 (55.8%)						
**Sleep duration WE**									
ADHD	49 (21.3%)	51 (22.2%)	130 (56.5%)	0.914	NS	24.650	**<0.001**	22.627	**<0.001**
ASD	17 (17%)	25 (25%)	58 (58%)						
Controls	78 (23.1)	26 (7.7%)	233 (69.2%)						
**Sleep latency WD**									
ADHD	103 (48.4%)	0 (0%)	110 (51.6%)	5.195	NS	7.190	**0.027**	12.058	**0.002**
ASD	41 (57.7%)	1 (1.4%)	29 (40.8%)						
Controls	116 (31.1%)	1 (0.3%)	196 (62.6%)						
**Sleep latency WD**									
ADHD	117 (51.6%)	2 (0.9%)	110 (48.0%)	4.181	NS	9.416	**0.009**	14.101	**<0.001**
ASD	49 (61.3%)	2 (2.5%)	29 (36.3%)						
Controls	127 (38.5)	7 (2.1%)	196 (59.4%)						

WD = weekday; WE = weekend. Significant differences at *p* < 0.05 are in bold.

**Table 7:** Comparison of sleep disorders before and during lockdown in the three groups.

					ADHD vs. ASD		ADHD vs. Control		ASD vs. Control	
		ADHD %	ASD %	CONT %	χ^2^	*p*	χ^2^	*p*	χ^2^	*p*
**Difficulties falling asleep**	before	64 (27.1%)	23 (23.0%)	72 (21.2%)	0.621	NS	2.727	NS	0.152	NS
	during	92 (39.0%)	35 (35.0%)	65 (19.1%)	0.474	NS	27.726	**<0.001**	11.099	**0.001**
**Anxiety at bedtime**	before	36 (15.3%)	12 (12.0%)	19 (5.6%)	0.607	NS	15.070	**<0.001**	4.831	**0.028**
	during	56 (23.7%)	22 (22.0%)	38 (11.2%)	0.118	NS	16.073	**<0.001**	7.687	**0.006**
**Hypnic jerks**	before	30 (12.7%)	11 (11.0%)	14 (4.1%)	0.192	NS	14.583	**<0.001**	6.830	**0.009**
	during	39 (16.5%)	12 (12.0%)	13 (3.8%)	1.117	NS	27.366	**<0.001**	9.640	**0.002**
**Rhythmic movement dis.**	before	10 (4.2%)	7 (7.0%)	5 (1.5%)	1.116	NS	4.204	**0.040**	8.906	**0.003**
	during	14 (5.9%)	9 (9.0%)	7 (2.1%)	1.037	NS	5.950	**0.015**	10.625	**0.001**
**Night awakenings >2**	before	29 (12.3%)	10 (10.0%)	11 (3.2%)	0.358	NS	17.667	**0.000**	7.780	**0.005**
	during	34 (14.4%)	18 (18.0%)	21 (6.2%)	0.693	NS	10.926	**0.001**	13.373	**0.000**
**Restless sleep**	before	80 (33.9%)	28 (28.0%)	75 (22.1%)	1.120	NS	9.928	**0.002**	1.521	NS
	during	83 (35.2%)	34 (34.0%)	71 (20.9%)	0.042	NS	14.517	**0.000**	7.318	**0.007**
**Snoring/apneas**	before	19 (8.1%)	8 (8.0%)	13 (3.8%)	0.000	NS	4.745	**0.029**	2.966	NS
	during	22 (9.3%)	9 (9.0%)	12 (3.5%)	0.009	NS	8.416	**0.004**	5.088	**0.024**
**Sleepwalking**	before	1 (0.4%)	3 (3.0%)	0 (0.0%)	-	NS *	-	NS *	-	**0.011** *
	during	14 (5.9%)	7 (7.0%)	8 (2.4%)	0.137	NS	4.858	**0.028**	5.068	**0.024**
**Sleep terrors**	before	3 (1.3%)	0 (0.0%)	4 (1.2%)	-	NS *	-	NS *	-	NS *
	during	4 (1.7%)	4 (4.0%)	2 (0.6%)	-	NS *	-	NS *	-	**0.026** *
**Bruxism**	before	39 (16.5%)	7 (7.0%)	31 (9.1%)	5.393	**0.020**	7.160	**0.007**	0.439	NS
	during	26 (11.0%)	11 (11.0%)	21 (6.2%)	0.000	NS	4.355	**0.037**	2.666	NS
**Nightmares**	before	28 (11.9%)	2 (2.0%)	16 (4.7%)	-	**0.003** *	10.118	**0.001**	-	NS *
	during	42 (17.8%)	5 (5.0%)	35 (10.3%)	9.560	**0.002**	6.771	**0.009**	2.621	NS
**Daytime sleepiness**	before	29 (12.3%)	4 (4.0%)	15 (4.4%)	-	**0.026** *	12.249	**0.000**	-	NS *
	during	47 (19.9%)	15 (15.0%)	27 (7.9%)	1.128	NS	17.839	**0.000**	4.459	**0.035**

Significant differences at *p* < 0.05 are in bold. * Fisher’s exact test was applied.

## Data Availability

This study was not preregistered, and the data that support the findings of this study are available on request from the corresponding author (O.B.). The data are not publicly available due to restrictions, e.g., containing information that could compromise the privacy of research participants.

## Supplementary Materials

The following are available online at https://www.mdpi.com/article/10.3390/brainsci11091139/s1, Supplementary Table S1. Sleep disorders before and during lockdown in the three groups. Figure S1: Sleep disorders in the three groups before lockdown, Figure S2: Sleep disorders in the three groups during lockdown.
