# Supplementary material for: Changes in Sleep Patterns and Disorders in Children and Adolescents with Attention Deficit Hyperactivity Disorders and Autism Spectrum Disorders during the COVID-19 Lockdown
Source: Brain Sci. 2021 Aug 27;11(9):1139. doi: 10.3390/brainsci11091139 (PMC8469743; doi:10.3390/brainsci11091139)
Supplement: Supplementary file 1 [file brainsci-11-01139-s001.zip › brainsci-1340297-supplementary.pdf]

SUPPLEMENTARY MATERIALS

Changes in sleep patterns and disorders in children and adolescents with Attention Deficit Hyperactivity Disorders and Autism Spectrum Disorders during the COVID-19 lockdown

Oliviero Bruni, MD[1*], Maria Breda, MD[2], Raffaele Ferri, MD[3], Maria Grazia Melegari, MD[1],

[1]*Department of Developmental and Social Psychology, Sapienza University, Rome, Italy*

[2]*Child Neurology and Psychiatry Unit, Department of Human Neurosciences, Sapienza University, Rome, Italy*

[3] *Sleep Research Centre, Department of Neurology IC, Oasi Research Institute - IRCCS, Troina, Italy*

Address correspondence to:

*Oliviero Bruni MD

Department of Social and Developmental Psychology, Sapienza University

Via dei Marsi 78 - 00185 Rome, Italy

Telephone number + 39-3356078964

email: oliviero.bruni@uniroma1.it

SUPPLEMENTARY MATERIAL

Supplementary Table S1. Sleep disorders before and during lockdown in the three groups.

		ADHD %	p	ASD %	p	CONT %	p
Difficulties falling asleep	***before***	**64 (27.1%)**	**0.001**	**23 (23.0%)**	**0.029**	72 (21.2%)	0.500
	during	**92 (39.0%)**		**35 (35.0%)**		65 (19.1%)	
Anxiety at bedtime	***before***	**36 (15.3%)**	**0.007**	**12 (12.0%)**	**0.006**	**19 (5.6%)**	**0.003**
	during	**56 (23.7%)**		**22 (22.0%)**		**38 (11.2%)**	
Hypnic jerks	*before*	30 (12.7%)	0.136	11 (11.0%)	1.000	14 (4.1%)	1.000
	during	39 (16.5%)		12 (12.0%)		13 (3.8%)	
Rhythmic movement dis.	*before*	10 (4.2%)	0.424	7 (7.0%)	0.625	5 (1.5%)	0.500
	during	14 (5.9%)		9 (9.0%)		7 (2.1%)	
Night awakenings >2	*before*	29 (12.3%)	0.500	10 (10.0%)	0.115	11 (3.2%)	0.052
	during	34 (14.4%)		18 (18.0%)		21 (6.2%)	
Restless sleep	*before*	80 (33.9%)	0.780	28 (28.0%)	0.327	75 (22.1%)	0.659
	during	83 (35.2%)		34 (34.0%)		71 (20.9%)	
Snoring/apneas	*before*	19 (8.1%)	0.453	8 (8.0%)	1.000	13 (3.8%)	1.000
	during	22 (9.3%)		9 (9.0%)		12 (3.5%)	
Sleepwalking	***before***	**1 (0.4%)**	**<0.001**	3 (3.0%)	0.125	**0 (0.0%)**	**<0.001**
	during	**14 (5.9%)**		7 (7.0%)		**8 (2.4%)**	
Sleep terrors	*before*	3 (1.3%)	1.000	0 (0.0%)	0.133	4 (1.2%)	0.625
	during	4 (1.7%)		4 (4.0%)		2 (0.6%)	
Bruxism	***before***	**39 (16.5%)**	**0.015**	7 (7.0%)	0.219	31 (9.1%)	0.052
	during	**26 (11.0%)**		11 (11.0%)		21 (6.2%)	
Nightmares	*before*	28 (11.9%)	0.065	2 (2.0%)	0.250	**16 (4.7%)**	**0.003**
	during	42 (17.8%)		5 (5.0%)		**35 (10.3%)**	
Daytime sleepiness	***before***	**29 (12.3%)**	**0.005**	**4 (4.0%)**	**0.003**	**15 (4.4%)**	**0.029**
	during	**47 (19.9%)**		**15 (15.0%)**		**27 (7.9%)**	

Significant differences at $p < 0.05$ are in bold.

Supplementary Figure S1. Sleep disorders in the three groups before lockdown.

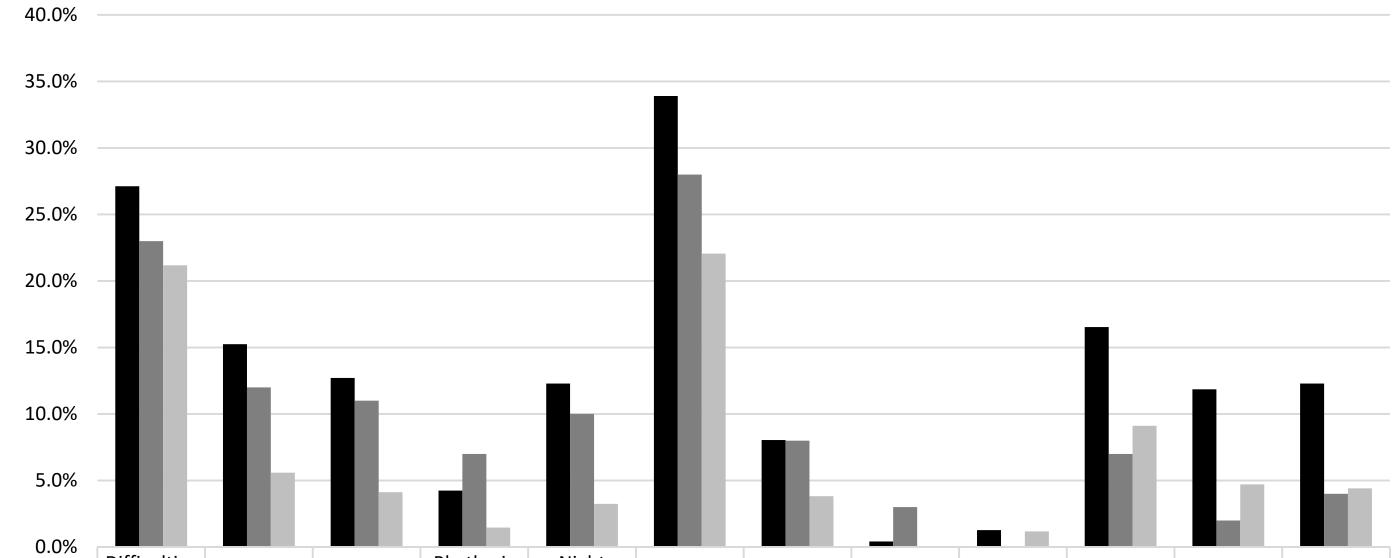

	Difficulties falling asleep	Anxiety at bedtime	Hypnic jerks	Rhythmic movement disorder	Night awakening s >2	Restless sleep	Snoring/ apneas	Sleepwalki ng	Sleep terrors	Bruxism	Nightmares	Daytime sleepiness
ADHD %	27.1%	15.3%	12.7%	4.2%	12.3%	33.9%	8.1%	0.4%	1.3%	16.5%	11.9%	12.3%
ASD %	23.0%	12.0%	11.0%	7.0%	10.0%	28.0%	8.0%	3.0%	0.0%	7.0%	2.0%	4.0%
CONT %	21.2%	5.6%	4.1%	1.5%	3.2%	22.1%	3.8%	0.0%	1.2%	9.1%	4.7%	4.4%

Supplementary Figure S2. Sleep disorders in the three groups during lockdown.

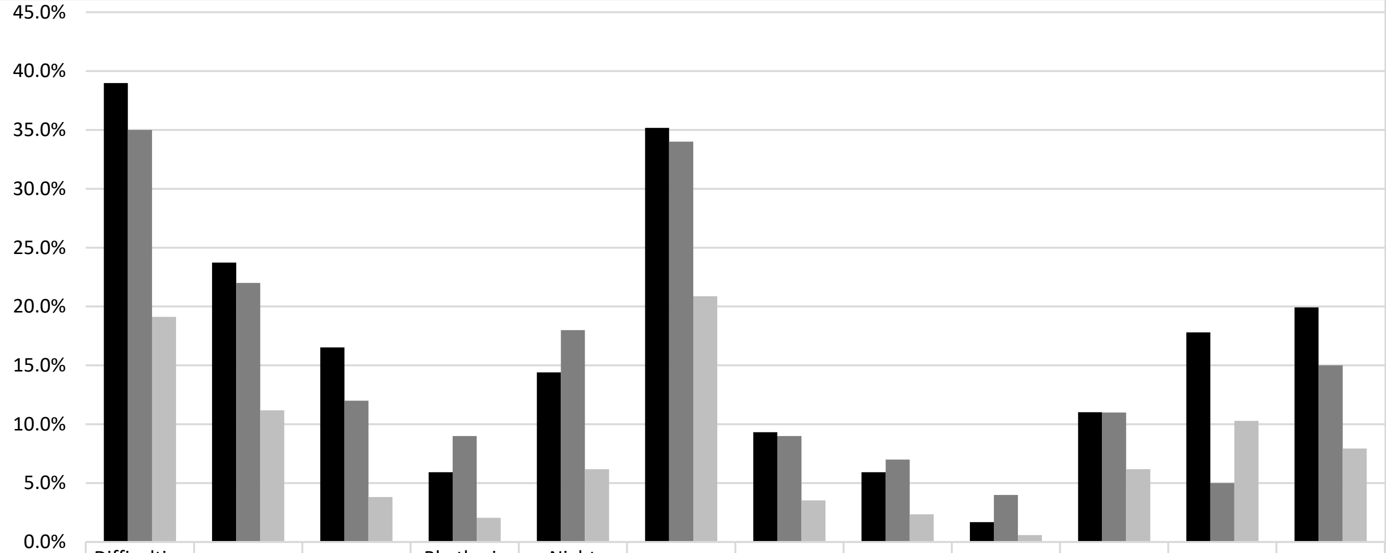

	Difficulties falling asleep	Anxiety at bedtime	Hypnic jerks	Rhythmic movement disorder	Night awakenings >2	Restless sleep	Snoring/ apneas	Sleepwalki ng	Sleep terrors	Bruxism	Nightmares	Daytime sleepiness
■ ADHD %	39.0%	23.7%	16.5%	5.9%	14.4%	35.2%	9.3%	5.9%	1.7%	11.0%	17.8%	19.9%
■ ASD %	35.0%	22.0%	12.0%	9.0%	18.0%	34.0%	9.0%	7.0%	4.0%	11.0%	5.0%	15.0%
■ CONT %	19.1%	11.2%	3.8%	2.1%	6.2%	20.9%	3.5%	2.4%	0.6%	6.2%	10.3%	7.9%
